# Sequencing technology status of *BRCA1/*2 testing in Latin American Countries

**DOI:** 10.1038/s41525-020-0126-3

**Published:** 2020-06-02

**Authors:** Angela R. Solano, Edenir I. Palmero, Lucía Delgado, Dirce M. Carraro, Rocío Ortíz-López, Claudia L. Carranza, Carlos Santamaria, Laura Cifuentes, Lilian E. Jara Sosa, Amanda E. Toland

**Affiliations:** 1 INBIOMED, Facultad de Medicina UBA/CONICET and Genotipificacion y Cancer Hereditario, Dto. de Analisis Clínicos, CEMIC, Buenos Aires, Argentina; 2 Molecular Oncology Research Center, Barretos Cancer Hospital and Barretos School of Health Science, Dr Paulo Prata, FACISB, Barretos, Brazil; 3 Unidad de Oncogenética, Hospital de Clínicas, Facultad de Medicina, Universidad de la República, Montevideo, Uruguay; 4 CIPE, International Research Center; A.C.Camargo Cancer Center, Sao Paulo, Brazil; 5 Tecnologico de Monterrey. Escuela de Medicina y Ciencias de la Salud, Monterrey, Mexico; 6 INVEGEM/ROZAS BOTRÁN ONG, Guatemala, Guatemala; 7 Hospital de Niños, Universidad de Costa Rica, San José, Costa Rica; 8 GIOD Group, Universidad Cooperativa de Colombia, Pasto, and Human Molecular Genetics Lab, Universidad del Valle, Cali, Colombia; 9 ICCBM, Facultad de Medicina, Universidad de Chile, Santiago, Chile; 10 Department of Cancer Biology and Genetics, Division of Human Genetics, Department of Internal Medicine, Comprehensive Cancer Center, the Ohio State University, Columbus, OH United States

**Keywords:** Next-generation sequencing, Cancer

The goal of this study was to survey laboratories in Latin America performing *BRCA1* and *BRCA2* (*BRCA1/2*) testing using the same questionnaire administered previously to other testing laboratories around the world^1^. The initial study only captured information from two laboratories in the Latin American region. As this region of the world was under-represented, this study aimed to fill in the knowledge gap.

A letter of invitation was sent to 12 laboratories directors of known academic centers in the region performing genetic testing. Two additional laboratories were identified, one at the XVII Congress of the Latin American Association of Genetics (ALAG) meeting in Mendoza, Argentina and a second at the 2019 American Society of Human Genetics meeting in Houston, TX, USA. Nine laboratories responded to the invitation (Fig. 1). We reached out to 14 laboratories in 11 countries. These laboratories were chosen as those we were aware of that performed completed in-house clinical grade sequencing of BRCA1 and BRCA2. Complete testing includes DNA isolation, sequencing, data analysis, and sending out clinical grade reports. Of reporting laboratories, there are many similarities across sites.

**Fig. 1:**
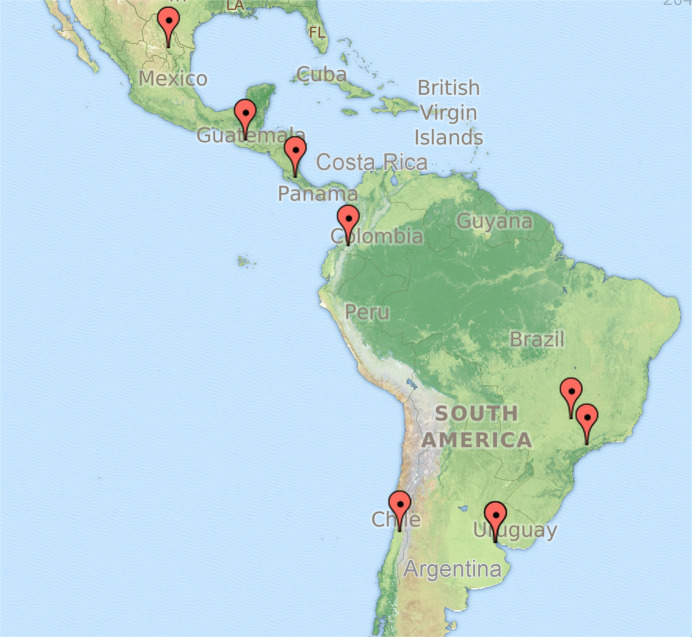
Location of the laboratories participating in this study. ZeeMap (© 1996–2005 ZeeMap, Map data: ©Google), which uses IP of origin from the surveyed laboratories, was used to draw the balloons that localize the position on Latin America. The marks for Buenos Aires and Montevideo were superimposed and appear as a single balloon.

Eight laboratories used one or more next-generation sequencing platforms to identify *BRCA1/2* variants whereas one used Sanger Sequencing. All laboratories sequenced all coding regions of *BRCA1/2*, but the intronic regions interrogated varied from 1–50 bp. Six of the nine laboratories also sequenced 5’ and 3’UTRs. The laboratories using NGS all conducted panel testing of which both *BRCA1/2* were evaluated. One laboratory included only *BRCA1/2* on its panel. In contrast, deletion/duplication analyses were done by seven of the nine laboratories with MLPA being the most commonly utilized method of detection.

Per quality-control metrics, all laboratories confirmed any pathogenic/likely pathogenic variants identified, primarily by Sanger sequencing. Five of the nine labs also confirmed variants of uncertain significance. The rate of VUS for *BRCA1/2* for the six labs reporting ranged from 1.24 to up to 60% with an average of 17.3% and a median of 9.5%. The average read depth for *BRCA1/BRCA2* by NGS was 1716X with a range of 200–10,000 reads. The minimum read depth for each lab to meet their quality guidelines ranged from 20 to 500 (average of 128).

An important function of clinical testing laboratories is how they evaluate variants of uncertain clinical significance. All nine laboratories performed variant classification in-house. Eight of the nine laboratories used the American College of Medical Genetics variant classification guidelines. One of these laboratories uses ACMG guidelines plus data from their own database that contains local and regional variant frequencies. One did not use ACMG and had its own annotation process. Most laboratories had a Board-Certified Molecular Geneticist responsible for variant classification whereas three used individuals with clinical genetics expertise and one used both genetic counselors and clinical genetics experts. Six of the nine laboratories shared their variant information with public databases including ClinVar (four), LOVD (three), BIC (four), and Global Alliance (one).

The laboratories reported a wide variety in the number of samples tested. Between October 2015 and September 2016 (the time period specifically surveyed) the range of samples for the eight laboratories reporting was from 20 to 999 with an average of 208. The turn-around time for results was a little bit over a month (average 35 days) with a range from 3–4 weeks to 2 months.

One important point to note is that only laboratories that did *BRCA1/2* genetic testing in-house and did not send samples to another reference laboratory were included in this study. One reason for excluding these laboratories was to be consistent with the original “worldwide” survey in which only laboratories that tested samples in-house were included in order to ensure detailed knowledge of the technological practices at each site. There are a few centers in Latin America that currently send DNA for sequencing to a reference laboratory for clinical or research grade testing, some outside of the country^2,3^.

Reference laboratories do not always share variants in public database or their algorithms for variant classification. Generating data from local and regional populations is an important factor for classification of genetic variants. Fortunately, a few reports describe genetic variant data from patients of the different countries in Latin America^4–13^. The collection and documentation of regional genetic variants are valuable in order to understand the genetic landscape of the region, which is under-represented in many United States or European variant databases. This information is critical as novel genetic variants are very frequently detected in most Latin American countries, and there may be regional founder pathogenic variants or common polymorphisms^6–16^.

One limitation of this study is that there was no representation from many Latin American countries. We now note that we had a 64% response rate and had representation from 40% of Latin American countries. Some countries may send the majority of their samples to laboratories outside of their country, but it is likely that we missed some key testing centers. Another limitation of this study is that the survey had a few questions on testing practices in 2016 such as turn-around-time and number of samples processed. Many of these laboratories have updated their practices since 2016, including increasing the number of samples analyzed in their group. To address this, one additional question on the number of expected samples to be tested for *BRCA1/2* was asked of the reporting laboratories (Table 1). The number of samples tested per year since the original reporting period has increased for most laboratories with a range of 40–1075 and an average of 123.

**Table 1:** Samples analyzed at the time of the survey and numbers expected for 2019.

Country	Laboratory	Expected samples 2019	Samples 2016–2017	Technology
Argentina	Genotipificacion y Cancer Hereditario, CEMIC	1075	999	Illumina
Brazil^1^	Molecular Oncology and School of Sciences, Barreto	100	50	Ion Torrent
Brazil^3^	CIPE, International Research Center; A.C.Camargo Cancer Center, Sao Paulo	300	510	Illumina
Chile	Facultad de Medicina, Universidad de Chile, Santiago, Chile	45	20	Illumina
Colombia	Universidad Cooperativa de Colombia	50 (ND)	NA	Illumina
Costa Rica	Hopital de Niños, San Jose de Costa Rica, CR	45	NA	Illumina
Guatemala	INVEGEM/ROZAS BOTRÁN ONG	140	110	Illumina
Mexico	Tecnologico de Monterrey. Escuela de Medicina y Ciencias de la Salud. Monterrey, MEXICO	90	50	Illumina
Uruguay	Functional Genomics Unit, Pasteur Institute of Montevideo	40	20	Illumina

ND: The service was implemented on a grant basis; the samples will be determined at the end of the year according the budget received; up to September 2019: 25 samples.

NA: The service was implemented on research basis and later was made available for diagnosis.

A recent report summarizes the various technologies in Argentina as a further support for the development reached in genetic and genomic medicine in the region^17^.

In summary, there are multiple laboratories in Latin America performing genetic testing for *BRCA1/2* pathogenic variants. These laboratories are using state-of-the art platforms with similar quality-control metrics and variant classification protocols as laboratories in Europe and other regions of the world.

## Supplementary Information

Supplementary Information

## Data Availability

All data generated or analysed during this study are included in this published article and its Supplementary Data Set 1.
