# Supplementary material for: Sequencing technology status of BRCA1/2 testing in Latin American Countries
Source: NPJ Genom Med. 2020 Jun 2;5:22. doi: 10.1038/s41525-020-0126-3 (PMC7265546; doi:10.1038/s41525-020-0126-3)
Supplement: Supplementary file 1 — Supplementary Information [file 41525_2020_126_MOESM1_ESM.pdf]

Does your laboratory perform clinical genetic testing of BRCA1/BRCA2 and/or other genes associated with heredi	Enter your Laboratory/Company Name - Click to write Choice 1
Yes	
Yes	Laboratorio de GenÃ©tica Molecular Humana
Yes	Genotipificacion y Cancer hereditario, DAC, CEMIC
Yes	
Yes	University Hospital
Yes	Laboratory of Genomics Diagnostic (LDG) at A.C.Camargo Cance
Yes	Tecnologico de Monterrey
Yes	National Children Hospital
Yes	INVEGEM

Enter your location (City/Country) - Click to write Choice 1 - Text
Colombia
Santiago/Chile
Ciudad AutÃ³noma de Buenos Aires, Argentina
Barretos - SÃ£o Paulo - Brazil
Montevideo - Uruguay
Sao Paulo/Brazil
Monterrey/MÃ©xico
San Jose, Costa Rica
GUATEMALA

What is the platform currently used by your laboratory for DNA sequencing of hereditary cancer risk genes? (Select all that apply). - Selected Choice
Sanger Sequencing
Illumina HiSeq
Illumina MiSeq,Illumina HiSeq,Ion Torent,Sanger Sequencing
Ion Torent,Sanger Sequencing
Illumina MiSeq
NextSeq,Other
Illumina MiSeq
Illumina MiSeq,Sanger Sequencing
Other

What is the platform currently used by your laboratory for DNA sequencing of hereditary cancer risk genes? (Select all that apply). - Other - Text
MiniSeq
Illumina Miniseq

What is the platform currently used by your laboratory for deletion/duplication analysis, when applicable, of hereditary cancer risk genes. (Select all that apply) - Selected Choic
Deletion/duplication analysis is not performed for any genes
MLPA
MLPA
MLPA
MLPA,Next-generation sequencing platform,Other
MLPA,Other
MLPA,Next-generation sequencing platform
Next-generation sequencing platform

What is the platform currently used by your laboratory for deletion/duplication analysis, when applicable, of hereditary cancer risk genes. (Select all that apply) - Other - Text
NGS Sophia Solution
PCR for small deletions

Which genes are not analyzed for deletion/duplication? - Selected Choice
We do not perform deletion/duplication analysis for any genes
We do not perform deletion/duplication analysis for any genes
The following genes do not have deletion/duplication analysis performed
Deletion/duplication analysis is performed for every gene we assess
We do not perform deletion/duplication analysis for any genes

The following genes do not have deletion/duplication analysis performed
The following genes do not have deletion/duplication analysis performed
Deletion/duplication analysis is performed for every gene we assess
The following genes do not have deletion/duplication analysis performed

Which genes are not analyzed for deletion/duplication? - The following genes do not have deletion/duplication analysis performed - Text

PMS2 and TP53

AIP, ALK, BAP1, BLM, BMPR1A, BUB1B, CDC73, CDK4, CDKN1C, CDKN2A, CEBPA, CEP57, CYLD, DDB2, DICER1, DIS3L2, EGFR, ERCC2, ERCC3, ERCC4, ERCC5, EXT1, EXT2, EZH2, FANCA,
We do deletion/duplication analysis only for BRCA genes

MSH6, STK11, TP53, CDH1

Are all coding exons covered for genes in your hereditary cancer multigene panels? (Note exceptions in next question). - Selected Choice
No (list genes that are not covered in next question)
Yes
Yes
Yes
Yes
Yes
Yes
Yes
Yes

Please describe any exons (and their affected gene) not covered in your multigene panels (e.g. only 1100delC assessed in CHEK2, no analysis of exons 12-15 in PMS2, single varia
Only 1100delC assessed in CHEK2. Only T241M assessed in XRCC3.

Are the following promoter regions or specialty results included when the applicable gene is part of the panel? (Check all that apply). - Selected Choice

Other
MSH2 promoter,MSH2 Boland inversion,Sequencing of exons 12-15 in PMS2
MSH2 promoter,Sequencing of exons 12-15 in PMS2

Sequencing of exons 12-15 in PMS2
MLH1 promoter

Are the following promoter regions or specialty results included when the applicable gene is part of the panel? (Check all that apply). - Other - Text

We don't analyze these regions

Are full intronic regions of any genes analyzed?
No
No
No
No
No
No
No
No
No

Are identified variants confirmed using another method (or the same method) before reporting? - Selected Choice
Yes. Both unclear and pathogenic/likely pathogenic variants are confirmed.
Yes. Only pathogenic/likely pathogenic variants are confirmed

Yes. Both unclear and pathogenic/likely pathogenic variants are confirmed.
Yes. Both unclear and pathogenic/likely pathogenic variants are confirmed.
Yes. Both unclear and pathogenic/likely pathogenic variants are confirmed.
Yes. Only pathogenic/likely pathogenic variants are confirmed
Yes. Both unclear and pathogenic/likely pathogenic variants are confirmed.
Yes. Only pathogenic/likely pathogenic variants are confirmed
Yes. Only pathogenic/likely pathogenic variants are confirmed

What methods/platforms are used to confirm identified genetic variants? (Check all that apply). - Selected Choice
Repeat sample analysis with original technology,Sanger Sequencing
Sanger Sequencing,MLPA (for deletion/duplication variants)
Sanger Sequencing,MLPA (for deletion/duplication variants)
Sanger Sequencing,MLPA (for deletion/duplication variants)
Sanger Sequencing
Repeat sample analysis with original technology,Sanger Sequencing,MLPA (for deletion/duplication variants),Other
Sanger Sequencing,MLPA (for deletion/duplication variants)
Sanger Sequencing,MLPA (for deletion/duplication variants)
Sanger Sequencing

What methods/platforms are used to confirm identified genetic variants? (Check all that apply). - Other - Text

using a second collected sample

How many base pairs of intronic regions are typically assessed? - Selected Choice
11-20 bp
11-20 bp
Other
6-10 bp
6-10 bp
11-20 bp
6-10 bp
11-20 bp
11-20 bp

How many base pairs of intronic regions are typically assessed? - Other - Text

1-50 bp and All previously established clinically significant intronic variants

What regulatory regions are included in analysis of BRCA1 and BRCA2? (check all that apply) - Selected Choice
3'UTR,5'UTR

Introns,3'UTR,5'UTR
Promoters,Introns,3'UTR,5'UTR
3'UTR,5'UTR

3'UTR,5'UTR,Other

3'UTR,5'UTR

What regulatory regions are included in analysis of BRCA1 and BRCA2? (check all that apply) - Other - Text

and only BRCA1 promoter

What is your analytic sensitivity of BRCA1/2? - Describe - Text

Unknown
97% - 100%
NA

Unknown
Not available
20 ng of DNA for NGS and 100 ng of DNA for Sanger
Unknown
Unknown

How many reference samples were used to determine BRCA1/2 analytic sensitivity? - Number - Text

15
60 approximately
NA
100

Not available
20 samples
Unknown
Unknown

What is your percentage of variants of uncertain significance (VUS) in BRCA1/2? - Selected Choice

Not calculated
Not calculated
Percentage
Percentage
Not calculated
Percentage
Percentage
Percentage
Percentage

What is your percentage of variants of uncertain significance (VUS) in BRCA1/2? - Percentage - Text

1.24
6%

9
15 al 20%
10
60%

How is the VUS rate calculated? - Selected Choice

Not calculated
Not calculated
Description

Not calculated
Description
Description
Description
Description

How is the VUS rate calculated? - Description - Text

207 VUS/16716 total variants

VUS per patient serie analyzed
Based on the number of studied cases. The variants are classified using the ACMG criteria.
number patients harboring VUS BRCA/number all analyzed patients
VUS/TOTAL OF VARIANTS FOUND

For next-generation sequencing technology, what is your average depth of base pair reads across all genes? - Selected Choice

Not applicable (NGS not performed)
Average number reads
Average number reads
Average number reads

Average number reads
Average number reads
Average number reads
Average number reads
Average number reads

eration sequencing technology, what is your average depth of base pair reads across all genes? - Average number reads - Text

800
500X
200 x
Above 700
300
The average is 250 x some times reaching up to 1,000X.
200
5000-15000

What is your minimum depth of base pair reads needed to meet your quality criteria? - Selected Choice
Not applicable (NGS not performed)
Minimum
Minimum
Minimum
Minimum
Minimum
Minimum
Minimum
Minimum

What is your minimum depth of base pair reads needed to meet your quality criteria? - Minimum - Text

20
50X
50 x
500
50
The minimum accepted depth is 50% for > 99% and 20X for 100%
50
for germline variants is 100

For next-generation sequencing technology, what is your average depth of base pair reads for BRCA1/2? - Selected Choice
Not applicable (NGS not performed)
Average Number reads BRCA1,Average number reads BRCA2
Average Number reads BRCA1,Average number reads BRCA2
Average Number reads BRCA1,Average number reads BRCA2
Average Number reads BRCA1,Average number reads BRCA2
Average Number reads BRCA1,Average number reads BRCA2
Average Number reads BRCA1,Average number reads BRCA2
Average Number reads BRCA1,Average number reads BRCA2
Average Number reads BRCA1,Average number reads BRCA2

ation sequencing technology, what is your average depth of base pair reads for BRCA1/2? - Average Number reads BRCA1 - Text

800
1000X
200 x
600
300
Similar than previous: 250X to 1,000X
200
5000-15000

For next-generation sequencing technology, what is your average depth of base pair reads for BRCA1/2? - Average number reads BRCA2 - Text

800
1000X
200 x
600
300
250X
200
5000-15,000

For BRCA1/2 what is your minimum depth of base pair reads? - Selected Choice

Not applicable (NGS not performed)

For BRCA1/2 what is your minimum depth of base pair reads? - Minimum depth BRCA1 - Text

20
100X
50 x
500
100
minimum read depth of 100 reads
50
100 for germline variants

For BRCA1/2 what is your minimum depth of base pair reads? - Minimum depth BRCA2 - Text

20
100X
50 x
500
100
Minimum read depth of 100
50
100 for germline variants

How are "low read" regions or gaps in sequencing data evaluated? (Check all that apply) - Selected Choice

Sanger sequence affected region
Sanger sequence affected region
Sanger sequence affected region
Sanger sequence affected region
No additional actions taken
Repeat entire assay,Other
No additional actions taken
Repeat entire assay
Repeat entire assay

How are "low read" regions or gaps in sequencing data evaluated? (Check all that apply) - Other - Text

NGS-based amplicon sequencing OR Repeat sequencing step

What is the analytic sensitivity of your multi-gene panels? - Selected Choice

Do not perform multi-gene panels
Do not perform multi-gene panels
Analytic sensitivity

Analytic sensitivity
Analytic sensitivity
Analytic sensitivity
Analytic sensitivity

What is the analytic sensitivity of your multi-gene panels? - Analytic sensitivity - Text

NA

100%
aprox 95 - 98%
No data
5% VF

What is the sensitivity and false discovery rate (FDR)/positive predictive value (PPV) for single nucleotide variants? - Sensitivity, FDR/PPV - Text

don't know this information
NA (errors discard by Sanger)

Not available
We do not calculate this value
No data

What is the sensitivity and FDR/PPV for indels? - Sensitivity, FDR/PPV - Text

don't know this information
NA (errors discard by Sanger)

Not available
Not evaluated
No data

What size indels can be reliably detected? - Selected Choice

Size
Size

Size
Depends on gene (please describe)
Size

What size indels can be reliably detected? - Size - Text

40 pb
15-20bp

Not available

1 to 50

What size indels can be reliably detected? - Depends on gene (please describe) - Text

We observed reduced confidence-reliability to detect insertions and deletions from 40 to 250 bp. It is possible to detect large deletions and insertions but the protocol is not focuse

Does your laboratory offer variant-specific testing for familial pathogenic variants found in multigene panels? - Selected Choice
Yes
No
Yes
Yes
Yes
Yes
Yes
Yes
Yes

In September 2016, what was your average turnaround time for tests of less than 10 genes? - Turnaround time - Text

1 - 2 months
60 days
30 - 45 days

30 working days
about 2 to 3 months
Not applicable (we did not assess NGS in 2016)
30 days

In September 2016, what was your average turnaround time for single syndrome tests, e.g. BRCA1/2? - Turnaround time - Text
1 Month
1 - 2 months
40 days
30 days
2 months
30 working days
3 or 4 weeks. This results were also used to determine preventive surgery
Not applicable (we did not assess NGS in 2016)
20 days

In September 2016, what was your average turnaround time for a multigene panel of more than 10 genes? - Turnaround time - Text

we donÂ´t apply
60 days

40 working days
Was about 4 to 6 months. By now the time is shorter, it is about 6 weeks turnaround time
Not applicable (we did not assess NGS in 2016)
35 days

Is variant interpretation performed by in-house staff? - Selected Choice
Yes
Yes
Yes
Yes
Yes
Yes
Yes
Yes
Yes

What variant interpretation guidelines do you follow? - Selected Choice
ACMG
In-house (describe)
Other
ACMG
ACMG
ACMG
ACMG
ACMG
ACMG

What variant interpretation guidelines do you follow? - In-house (describe) - Text

Using different data base

What variant interpretation guidelines do you follow? - Other - Text

ACMG and the laboratory has its own database representing the local and regional frequencies

Please describe your variant analysis process. Feel free to include a link if a detailed summary is available on your website. Please specify if there is a different process for BRCA

After variants are identified, annotation attributes such as genomic features, gene symbols, exonic functions, and amino acid changes are attached to the variant list. The annotatior
The obtained readings are processed and aligned with the reference genome hg19 using the Burrows Wheeler Aligner (BWA). Duplications reads are eliminated with Picard, and the

To evaluate a variant for clinical signification we review information that includes, but not limited to, the following: 1.Clinically significant databases: a.ClinVar (https://www.ncbi.nlm.nih.gov/clinvar/)
All the variant analysis go through the same process
FASTQ-SAM-BAM-VCF-Annotation (ClinVar, HGMD, Cosmic, OMIM, ExAc, 1000G, Swift polyphen for SNV). No difference between BRCA assessment or multi-gene studies
we used sophia genetics platform

How often does your laboratory re-assess previous variant results? - Selected Choice
Less than a year
Not applicable. Our laboratory does not re-assess previous variant classification
Less than a year
Less than a year
Re-assessment is done on an Ad hoc basis
Re-assessment is done on an Ad hoc basis
Less than a year
Not applicable. Our laboratory does not re-assess previous variant classification
Between 1-3 years

How often does your laboratory re-assess previous variant results? - Less than a year - Text

and Re-assessment is done on an Ad hoc basis

Is the ordering provider contacted when an unclear variant is reclassified? - Selected Choice
Yes, whether downgraded to benign/likely benign or upgraded to pathogenic/likely pathogenic
Yes, whether downgraded to benign/likely benign or upgraded to pathogenic/likely pathogenic
Yes, whether downgraded to benign/likely benign or upgraded to pathogenic/likely pathogenic
Yes, whether downgraded to benign/likely benign or upgraded to pathogenic/likely pathogenic
Yes, but only if reclassified as clinically actionable (e.g. pathogenic or likely pathogenic)
Yes, whether downgraded to benign/likely benign or upgraded to pathogenic/likely pathogenic
Yes, whether downgraded to benign/likely benign or upgraded to pathogenic/likely pathogenic
Yes, but only if reclassified as clinically actionable (e.g. pathogenic or likely pathogenic)
Providers are not automatically contacted

Who is involved with the variant classification process? (Check all that apply) - Selected Choice
Board Certified Molecular Geneticist
Board Certified Molecular Geneticist
Individuals with clinical genetics expertise on the specific genes being studied
Board Certified Molecular Geneticist,Board Certified Medical Geneticist,Individuals with clinical genetics expertise on the specific genes being studied
Genetic Counselor,Individuals with clinical genetics expertise on the specific genes being studied,Other
Individuals with clinical genetics expertise on the specific genes being studied
Board Certified Molecular Geneticist,Board Certified Medical Geneticist
Individuals with clinical genetics expertise on the specific genes being studied
Board Certified Molecular Geneticist

Do you offer variant-specific testing for family members when a VUS is identified for segregation and/or research studies (i.e. family studies)? - Selected Choice
No
No
No
Yes
Yes

No
Yes
No
No

If you offer family studies for VUS, please describe the process, including the required medical information, cost to patient, and turnaround time. - Description of family studies f

After being informed, the individual tested could propose to a relative the opportunity to be analyzed

It is done in women with some affected relative. They are invited to look for the mutation The costs are covered by foundations or Popular Insurance for 3 family members who war

Who is involved in report writing? (Check all that apply) - Selected Choice
Board Certified Molecular Geneticist,Genetic Counselor
Board Certified Molecular Geneticist
Individuals with clinical genetics expertise on the specific genes being studied
Board Certified Molecular Geneticist,Individuals with clinical genetics expertise on the specific genes being studied
Individuals with clinical genetics expertise on the specific genes being studied
Individuals with clinical genetics expertise on the specific genes being studied
Board Certified Molecular Geneticist,Board Certified Medical Geneticist
Individuals with clinical genetics expertise on the specific genes being studied
Board Certified Molecular Geneticist

Which public variant databases do you contribute data? (Check all that apply). - Selected Choice
LOVD,BIC
ClinVar,BIC
ClinVar,LOVD
Global Alliance
ClinVar,LOVD,BIC,Our laboratory does not contribute variant data to public databases
ClinVar,LOVD,BIC
Our laboratory does not contribute variant data to public databases
Our laboratory does not contribute variant data to public databases
Our laboratory does not contribute variant data to public databases

Which public variant databases do you contribute data? (Check all that apply). - Our laboratory does not contribute variant data to public databases - Text

Contribution to ClinVar and LOVD will be performed in next months

Do you respond to inquires about variants found in other laboratories? - Selected Choice
Yes
Yes
Yes
Yes
No
Yes
No
Other
No

Do you respond to inquires about variants found in other laboratories? - Other - Text

Not applicable

How many index cases for hereditary cancer risk were analyzed in your laboratory between October 2015-September 2016? - Index case number: - Text

20
999 cases
50
20
210
50
Not applicable (we did not assess NGS in 2016)
110

How many dedicated staff (full time equivalents) work at your company? - Staff size: - Text

3 people
5
4
3
4
4
2
16

Does your lab/company have board certified genetic counselors on staff? - Selected Choice
Yes
No
Yes
Our country does not have genetic counselors but we have an equivalent position (described)
Our country does not have genetic counselors but we have an equivalent position (described)
Other
Yes
Our country does not have genetic counselors but we have an equivalent position (described)
No

Does your lab/company have board certified genetic counselors on staff? - Our country does not have genetic counselors but we have an equivalent position (described) - Text

clinical medical geneticist
MD trained in oncogenetics

Clinical oncologist with training in genetic counseling

Does your lab/company have board certified genetic counselors on staff? - Other - Text

04 Oncogeneticists in A.C.Camargo Cancer Center (not in the lab)

How many genetic counselors or similar positions do you have on staff? - Number: - Text
1

4 in the company
5
3

2
2

Describe the various roles genetic counselor (or equivalent positions) have within the lab/company. (Check all that apply). - Selected Choice
Direct patient education (e.g. telephone genetic counseling or availability to answer questions,Direct medical provider education (e.g. face to face or telephone contact for clinical q

Other
Direct patient education (e.g. telephone genetic counseling or availability to answer questions,Direct medical provider education (e.g. face to face or telephone contact for clinical q
Direct patient education (e.g. telephone genetic counseling or availability to answer questions,Direct medical provider education (e.g. face to face or telephone contact for clinical q

Direct medical provider education (e.g. face to face or telephone contact for clinical questions),Educational lectures for the lay public,Educational lectures for healthcare providers,V

Direct patient education (e.g. telephone genetic counseling or availability to answer questions,Direct medical provider education (e.g. face to face or telephone contact for clinical q

Describe the various roles genetic counselor (or equivalent positions) have within the lab/company. (Check all that apply). - Other - Text

Counseling

In your country, is there only one reference laboratory that performs all of the clinical genetics testing for hereditary cancer syndromes? - Selected Choice

No

No

No

No

No

Yes

Are there any guidelines (national or regional) used to determine whether or not an ordered test is appropriate? - Selected Choice

No. We depend on the ordering clinician to determine appropriateness of testing

No. We depend on the ordering clinician to determine appropriateness of testing

Yes, we use the following guideline(s):

No. We depend on the ordering clinician to determine appropriateness of testing

No. We depend on the ordering clinician to determine appropriateness of testing

Yes, we use the following guideline(s):

No. We depend on the ordering clinician to determine appropriateness of testing

Yes, we use the following guideline(s):

No. We depend on the ordering clinician to determine appropriateness of testing

Are there any guidelines (national or regional) used to determine whether or not an ordered test is appropriate? - Yes, we use the following guideline(s): - Text

Instituto Nacional del CÃ¡ncer

Brazilian National Regulatory Agency for Private Health Insurance and Plans (ANS) criterias to benefit from genetic tests

local guidelines from Social security (public health)

Does your country provide guidelines for which genes can be included on clinical tests for hereditary cancer syndromes? (e.g. BRCA1 and BRCA2 only for hereditary breast and c

No

No

Yes, but this is only a recommendation (describe recommendation)

No, but only defined genes are covered by insurance

No

Yes, but this is only a recommendation (describe recommendation)

No

Yes. Only defined genes are allowed to be offered (describe)

No

Does your country provide guidelines for which genes can be included on clinical tests for hereditary cancer syndromes? (e.g. BRCA1 and BRCA2 only for hereditary breast and c

Brazilian National Regulatory Agency for Private Health Insurance and Plans (ANS) criterias to benefit from genetic tests

Are you able to bill private health insurance for tests (assuming patients meet appropriate criteria)? - Selected Choice

Yes

No. Patient is self-pay

Yes

Yes

Not applicable
Yes
Yes
Not applicable
Other

Are you able to bill private health insurance for tests (assuming patients meet appropriate criteria)? - Other - Text

both, testing is billed through national health systems and sometimes the patient pay it

Do you provide pre-verification services for insurance coverage?
Not applicable
No
Yes, once a sample is received OR if requested prior to sample submission
Yes, once a sample is received
Not applicable
Yes, once a sample is received OR if requested prior to sample submission
Not applicable
Not applicable
No

Do you offer financial assistance options? - Selected Choice

No
No
No
No
No

Do you offer genetic testing of DNA from buccal samples? - Selected Choice
No
No
Yes
Yes
No
Other
No
No
No

Do you offer genetic testing of DNA from buccal samples? - Other - Text

Saliva OR Blood

Do you offer genetic testing on skin fibroblasts? - Selected Choice
No
No
No
No
No
No
No
No
No

Do you offer genetic testing internationally? - Selected Choice

No
No
Other
Yes
No
No
No
No
No

Do you offer genetic testing internationally? - Other - Text

Ocassionally we recieved orders from neighbor countries and less frequently from USA

If international testing is available, do you cover shipping costs to submit samples? - Selected Choice

Yes
